# Relationship between monounsaturated fatty acids and sarcopenia: a systematic review and meta-analysis of observational studies

**DOI:** 10.1007/s40520-023-02465-0

**Published:** 2023-06-21

**Authors:** Sawan Ali, Graziamaria Corbi, Alessandro Medoro, Mariano Intrieri, Giovanni Scapagnini, Sergio Davinelli

**Affiliations:** 1 Department of Medicine and Health Sciences “V. Tiberio”, University of Molise, Via De Sanctis 1, Campobasso, Italy; 2 Department of Translational Medical Sciences, University of Naples “Federico II”, Naples, Italy

**Keywords:** Monounsaturated fatty acids, MUFA, Sarcopenia, Meta-analysis, Observational study

## Abstract

Accumulating evidence suggests that fatty acids (FAs) play an essential role in regulating skeletal muscle mass and function throughout life. This systematic review and meta-analysis aimed to examine the relationship between dietary or circulatory levels of monounsaturated FAs (MUFAs) and sarcopenia in observational studies. A comprehensive literature search was performed in three databases (PubMed, Scopus, and Web of Science) from inception until August 2022. Of 414 records, a total of 12 observational studies were identified for this review. Ten studies were meta-analysed, comprising a total of 3704 participants. The results revealed that MUFA intake is inversely associated with sarcopenia (standardized mean difference = − 0.28, 95% CI − 0.46 to − 0.11; *p* < 0.01). Despite the limited number of studies, our results suggest that lower MUFA intake is associated with a higher risk of sarcopenia. However, the available evidence is still insufficient and further investigations are needed to demonstrate this relationship.

**Supplementary Information:**

The online version contains supplementary material available at 10.1007/s40520-023-02465-0.

## Introduction

Sarcopenia is a progressive skeletal muscle disorder characterized by an accelerated decrease in muscle mass, muscle strength, and physical function [1, 2]. The prevalence of sarcopenia ranges from 10 to 27% in people over 60 years of age [3]. Elderly people suffering from sarcopenia are at high risk of adverse outcomes such as falls, fractures, poor quality of life, physical disability, hospitalization, and mortality [4, 5]. Since the aging population is increasing globally, sarcopenia has become a major public health issue [3]. In the past decade, sarcopenia has become the focus of intense investigations to better understand its pathophysiology and to identify effective preventive and treatment strategies [6, 7].

Besides insufficient protein intake and physical inactivity, accumulating evidence suggests that oxidative stress and inflammation are the main contributors to the complex etiology of sarcopenia [8, 9]. During aging, excessive production of reactive oxygen species (ROS), which controls the redox signal pathway, results in oxidative damage and mitochondrial dysfunction, impaired protein homeostasis, and eventually skeletal muscle dysfunction [10–12]. Age-related impairments in mitochondrial function may also increase oxidative stress and enhance the loss of proteostasis [9]. In addition, chronic low-grade inflammation during aging affects the metabolism of skeletal muscle cells through interactions among various cytokines [13]. Furthermore, inflammation causes oxidative stress and anabolic resistance, leading to the loss of muscle mass, strength, and function [13].

Although the association between diet and sarcopenia is still under investigation, a growing body of evidence suggests that nutrition has an important role in both the prevention and treatment of sarcopenia, in particular, a dietary pattern that ensure an adequate protein intake [14]. In addition, higher intakes of antioxidant nutrients play an important role in the regulation of muscle mass and function throughout life [15]. Indeed, current observational and clinical data show that increasing fruit and vegetable consumption could be effective tools to prevent or treat sarcopenia, especially by enhancing muscle strength and function [16]. Monounsaturated fatty acids (MUFA) have recently attracted considerable attention because of their role in health and disease prevention. MUFA are dietary nutrients most commonly found in olive oil, nuts, seeds, and some animal-based foods [17]. The most common MUFA in the diet is oleic acid (C18:1n-9, OL), which is also the highest MUFA provided in the diet (around 90% of all MUFA), followed by palmitoleic acid (16:1n-7, PLA) and vaccenic acid (18:1n-7) [17]. Detailed studies have shown that olive oil, the main source of MUFA, may attenuate oxidative stress, the main mechanism of sarcopenia pathogenesis [18–20]. Additionally, the Mediterranean diet, which is particularly rich in MUFA, reduces inflammation related to aging [21, 22].

Previous observational studies have evaluated the effects of MUFA on muscle parameters and sarcopenia, however, inconclusive results are reported. Therefore, the purpose of this systematic review and meta-analysis was to evaluate the association between dietary intake of MUFA and sarcopenia. In addition, we investigated whether this association is influenced by population characteristics, methods for MUFA assessment, and the diagnostic criteria of sarcopenia.

## Materials and methods

This systematic review and meta-analysis were performed according to the Preferred Reporting Items for Systematic Reviews and Meta-Analyses (PRISMA) guidelines [23].

### Eligibility criteria

Studies were eligible for this systematic review if they met the following criteria: (1) observational studies (cohort, case–control, cross-sectional) that evaluated the association between dietary or circulatory MUFA levels with sarcopenia, determined as confirmed/severe sarcopenia, probable sarcopenia, sarcopenic index, or sarcopenia risk score; (2) included adult people (aged 18 years and more); and (3) studies published in English. On the contrary, the exclusion criteria included: (1) randomized controlled trials; and (2) studies with secondary data (conference abstracts, meta-analyses, reviews, letters, and case reports). The PICOS criteria (i.e., participants, interventions, comparisons, outcomes, and study design) used to define the research question are shown in Table 1.

**Table 1:** PICOS criteria for inclusion of studies

Parameter	Description
Participants	People aged ≥ 18 years with and without sarcopenia
Intervention/exposure	Dietary or circulatory MUFA
Comparison	People with vs without sarcopenia
Outcome	Confirmed/severe sarcopenia, probable sarcopenia, sarcopenic index, sarcopenia risk score
Study design	Observational studies (cohort, cross-sectional, case–control)

*MUFA* monounsaturated fatty acids

### Search strategy

A systematic search was performed using three databases: PubMed, Scopus, and Web of Science. The following keywords and Boolean operators were used for the literature search: ("monounsaturated fatty acid" OR "monounsaturated fat" OR "MUFA" OR "palmitoleic acid" OR "oleic acid") AND ("sarcopenia" OR "sarcopenic" OR "muscular atrophy" OR "muscle strength" OR "muscle mass" OR "muscle fatigue" OR "physical performance"). At the same time, similar queries were respectively used for controlled vocabulary search: “monounsaturated fatty acids” [Mesh] AND “sarcopenia” [Mesh], INDEX TERMS “monounsaturated fatty acids” AND “sarcopenia”. The search was conducted from inception to August 2022. The search queries used to search in PubMed, Scopus, and Web of Science databases are presented in Table S1.

### Study selection and data extraction

**Fig. 1:**
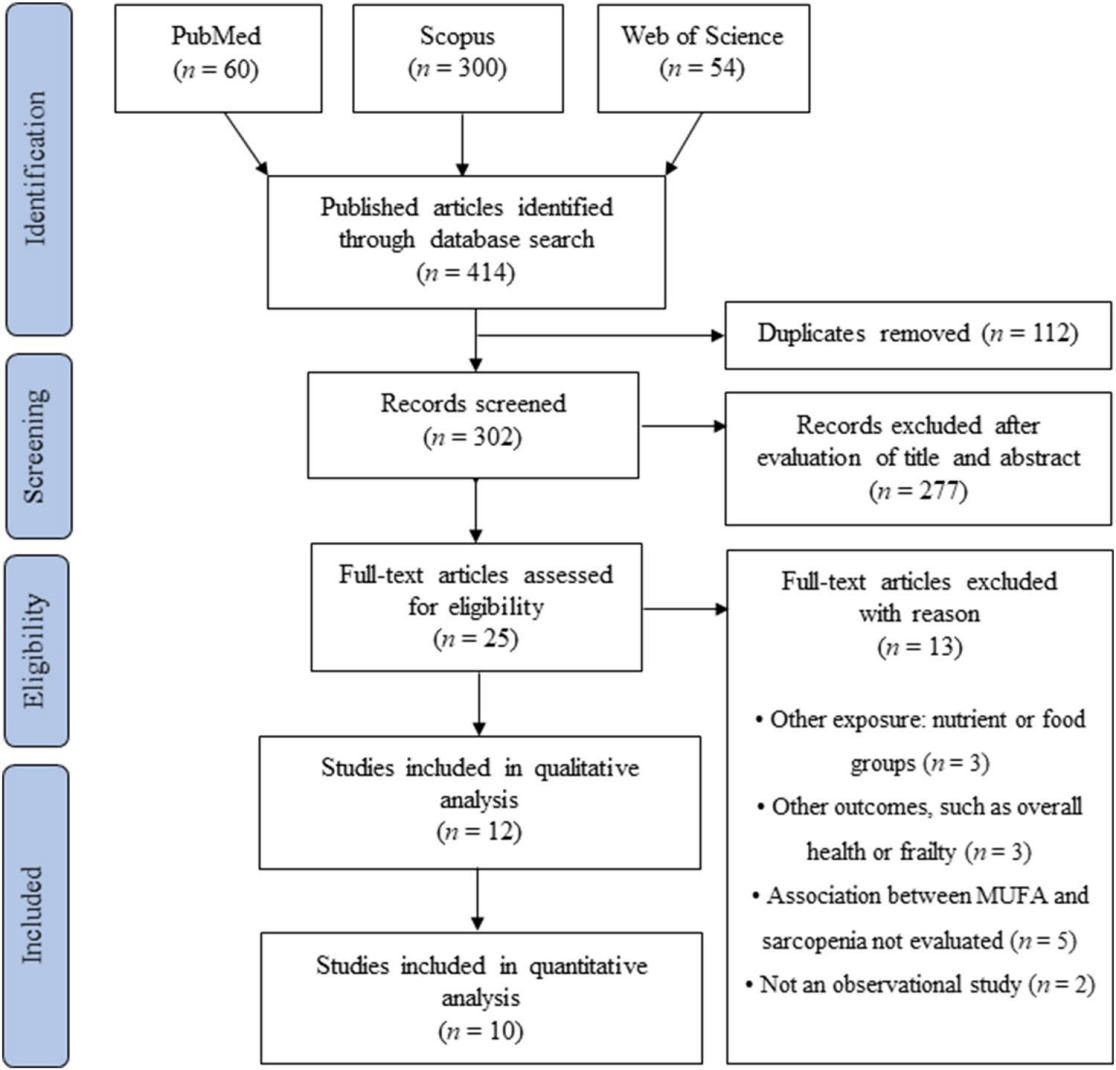
PRISMA flow diagram

After removing duplicate records with the reference management software EndNote X9 (Clarivate Analytics, Philadelphia, PA, USA), titles and abstracts of retrieved articles were screened for eligibility by two researchers (SA and SD). If an abstract did not provide enough information for evaluation, the full text was retrieved. Disagreements were resolved by a third reviewer (GC). Articles that met the eligibility criteria were selected for inclusion in the final review (Fig. 1). A data extraction table for the included studies was then developed. The following information was extracted: the first author (along with the year of publication and country of the study), study design, participant characteristics (sample size, age, gender), exposure assessment, outcome measure and sarcopenia definition, and the main findings.

### Quality assessment

Methodological quality assessments of the included studies were performed using the Newcastle–Ottawa Scale (NOS) for observational studies [24]. The NOS assesses three quality domains (selection, comparability, and outcome) divided into eight specific items. The NOS gives a maximum score of 9 points. Studies with NOS scores of 0–3, 4–6, and 7–9 were considered low, moderate and high quality, respectively [25].

### Statistical analysis

The meta-analysis was conducted using the R Software, version 4.0.3 (R Foundation for Statistical Computing, Vienna, Austria) and the interface RStudio version 1.4.1717 (R studio, PBC, Boston, MA, USA). Mean values and standard deviations (SD) of MUFA levels were extracted for the meta-analysis. The standardized mean difference (SMD) was used as a summary statistic because the studies assessed the outcome using different techniques and tools. A *p*-value of less than 0.05 was considered to be statistically significant. Heterogeneity among studies was examined with I^2^ statistics [26]. Random-effects models were used for the analyses and the possibility of small study effects was assessed qualitatively by a visual estimate of the funnel plot and quantitatively by calculation of the Egger and Begg’s tests [27]. In addition, subgroup analyses were performed that included consideration of sarcopenic obesity, participants' age (< 65 and ≥ 65 years to evaluate the aging effect), gender (men, women, and both), sarcopenia classification (confirmed/severe sarcopenia and probable sarcopenia), sarcopenia diagnostic criteria (the European Working Group on Sarcopenia (EWGSOP) [6], EWGSOP2 [7], the Asian Working Group for Sarcopenia (AWGS) [28], and the other diagnostic criteria), MUFA exposure assessment (dietary and circulatory), and MUFA intake unit (percent of total energy, percent of total FA, grams/day, MUFA/saturated FA (SFA) ratio).

## Results

### Study identification and selection

The PRISMA flow diagram of this systematic review and meta-analysis is presented in Fig. 1. A total of 414 potentially relevant articles (PubMed: 60, Scopus: 300, and Web of Science: 54) were yielded during initial literature searches. After eliminating 112 duplicated articles, 302 articles were screened by titles and abstracts. Eventually, a total of 12 articles were identified to be included in our study [29–40].

### Study characteristics

The main characteristics of the included studies are presented in Table 2. The studies were published between 2006 and 2021. The countries in which the studies were conducted were Spain [29, 31], Brazil [32, 33], Japan [36, 38], Canada [30], Iran [34], Finland [35], the Netherlands [39], and UK [40], and one study was conducted in multiple European countries [37]. All studies included both men and women, except for one study that included only men [35] and one that included only women [30]. The sample size ranged from 22 to 1535 subjects. The diagnostic criteria for sarcopenia were the EWGSOP [31], the EWGSOP2 [33–35, 37, 39], the AWGS [36, 38], and other diagnostic criteria [29, 30, 32, 40]. Seven of the included studies diagnosed confirmed/severe sarcopenia [31, 33, 35, 36, 38–40], and two studies determined probable sarcopenia among the participants [34, 35]. Additionally, Abete 2019 calculated sarcopenic index [29]. The sarcopenic index in this study was estimated by dividing the amount of appendicular skeletal muscle mass (kg) by body weight (kg) × 100. The authors then categorized the participants according to sex-specific sarcopenic index tertiles, providing an estimate of the amount of skeletal muscle mass relative to body size, where a higher index implied better health. Furthermore, Montiel-Rojas 2020 determined a continuous clustered sarcopenia risk score, which was estimated based on skeletal muscle mass index (SMI) and handgrip strength [37]. The authors first calculated sex-specific standardized values of SMI and handgrip strength and averaged them into composite z-scores, where higher scores implied a higher sarcopenia risk.

**Table 2:** Characteristics of the included studies

Study (author, year, country, reference)	Design	Participant characteristics	Assessment of MUFA levels	Outcome measure	Sarcopenia definition	Main findings
Abete (2019), Spain [29]	Cross-sectional from the PREDIMED-Plus trial	1535 subjects (mean age 65.2 years) 48% women	143-item semi-quantitative FFQ	Skeletal muscle mass index by DXA	Sarcopenic index tertiles (women: T1: < 21.0% and T3: ≥ 22.7; men: T1: < 26.3% and T3: ≥ 28.5%)	No difference in dietary MUFA levels between tertiles of sex-specific sarcopenic index
Aubertin-Leheudre (2006), Canada [30]	Cross-sectional	22 obese postmenopausal women (mean age 66 y)	3-day dietary record	Muscle mass index by DXA	Muscle mass index < 14.30 kg fat-free mass/m^2^	No difference in dietary MUFA levels between sarcopenic-obese and non-sarcopenic-obese subjects
Bibiloni (2018), Spain [31]	Cross-sectional	380 subjects (age range 55–80 years) 54% women	24-h dietary recall	Muscle strength by HGS test and physical performance by 8-f TUG test	EWGSOP	Significant lower MUFA levels in subjects with low HGS compared to those normal HGS (*p* = 0.001)
de-Franca (2020), Brazil [32]	Cross-sectional from ISA-Capital 2015 and 2015 ISA-Nutrition studies	218 community-dwelling adults (mean age 63 years) 52% women	Dietary recall questionnaires	Muscle mass by DXA, muscle strength by HGS test, and physical function by 4-m walking test	Muscle mass < 0.789 for men and < 0.512 for women^a^	No difference in dietary MUFA levels between osteo-sarcopenic obese and normal subjects
Dos Reis (2021), Brazil [33]	Cross-sectional	125 kidney transplant patients (mean age 48 y) 32% women	24-h dietary recall	Appendicular muscle mass by BI, muscle strength by HGS test, and physical performance by 4-m walking test	EWGSOP2	Significant lower MUFA levels in sarcopenic subjects compared to non-sarcopenic subjects (*p* = 0.005)
Esmaeily (2021), Iran [34]	Cross-sectional	201 community-dwelling older adults (mean age 65.72 years) 77% women	147-item semiquantitative FFQ	Muscle strength by HGS test	Prosarcopenia according to EWGSOP2	Higher ratio of MUFA and PUFA to SFA in subjects with a low prosarcopenia scores compared to high prosarcopenia scores (*p* = 0.03)
Jyväkorpi (2020), Finland [35]	Cross-sectional from HBS study	126 men (mean age 87 y)	3-day food diary	Appendicular muscle mass, muscle strength, and physical performance by SPPB	EWGSOP2	Significant negative association between MUFA levels and sarcopenia (*p* = 0.01)
Katoh (2020), Japan [36]	Retrospective cross-sectional study	308 cardiovascular patients (mean age 72 y) 43.5% women	Serum MUFA levels	Muscle mass by BI, muscle strength by HGS test, and physical performance by gait speed test	AWGS	Low serum levels of OL (*p* = 0.005) and high serum levels of NA (*p* < 0.001) and erucic acid (*p* < 0.001) in sarcopenic subjects compared to non-sarcopenic subjects
Montiel-rojas (2020), European countries [37]	Cross-sectional from NU-AGE Cohort	986 older adults (age range 65–79 years) 58% women	7-day food record	Skeletal muscle mass by DXA and muscle strength by HGS test	Sarcopenia risk score according to EWGSOP2	No significant association between MUFA levels and sarcopenia risk score
Otsuka (2021), Japan [38]	Cross-sectional from the fifth survey of the ROAD Study	1345 community-dwelling subjects (mean age 71.2 years) 67.5% women	BDHQ	Muscle mass by BI, muscle strength by HGS test, and physical performance by 6-m walking test	AWGS	Significant lower MUFA levels in sarcopenic subjects compared to non-sarcopenic subjects (*p* = 0.01)
ter Borg (2018), the Netherlands [39]	Cross-sectional Maastricht Sarcopenia study	227 older adults (age ≥ 65 years) 52% women	Blood MUFA levels	Skeletal muscle mass by BI, muscle strength by HGS test, and physical performance by gait speed test and chair stand test	EWGSOP	No significant association between MUFA levels and sarcopenia
Verlaan (2017), UK [40]	Case–control from PROVIDE study	132 older adults (mean age 71 years) 39% women	3-day food diary	Appendicular muscle mass by DXA, muscle strength by HGS test, and muscle function by SPPB	SPPB score of 4–9, muscle mass index < 37% for men and < 28% for women, and BMI 20–30 kg/m^2^	No significant difference in MUFA levels between sarcopenic and non-sarcopenic subjects

*FFQ* food frequency questionnaires, *DXA* dual-energy X-ray absorptiometry, *MUFA* monounsaturated fatty acids, *HGS*, handgrip strength, *8-f TUG* eight-foot time up-and-go, *EWGSOP* the European working group on sarcopenia in older people, *BI* bioimpedance, *EWGSOP2* revised EWGSOP, *PUFA* polyunsaturated fatty acids, *SFA* saturated fatty acids, *SPPB* short physical performance battery, *AWGS* the Asian working group for sarcopenia, *OL* oleic acid, *NA* nervonic acid, *BDHQ* brief-type diet history questionnaire, *BMI* body mass index

^a^The values < 0.789 for men and < 0.512 for women were used in the De-franca 2020 as cutpoints for low muscle mass based on the Foundation for the National Institutes of Health Sarcopenia Project

Two studies measured circulatory MUFA levels [36, 39], whereas the remaining ten studies estimated dietary intake of MUFA. The dietary MUFA were determined by food-frequency questionnaire (FFQ) [29, 34], 24-h dietary recall [31, 33], 3-day food diary [35, 40], 7-day food diary [37], dietary recall questionnaires [32], and brief-type self-administered diet history questionnaire (BDHQ) [38].

### Quality of the included studies

The quality of the included studies, based on NOS quality assessment, is shown in Table S2. One study achieved NOS scores of 6 (moderate quality) [30], and 11 studies was rated as “high” quality (7–9 points) [29, 31–38, 40].

### Meta-analysis of the effects of MUFA on sarcopenia

**Fig. 2:**
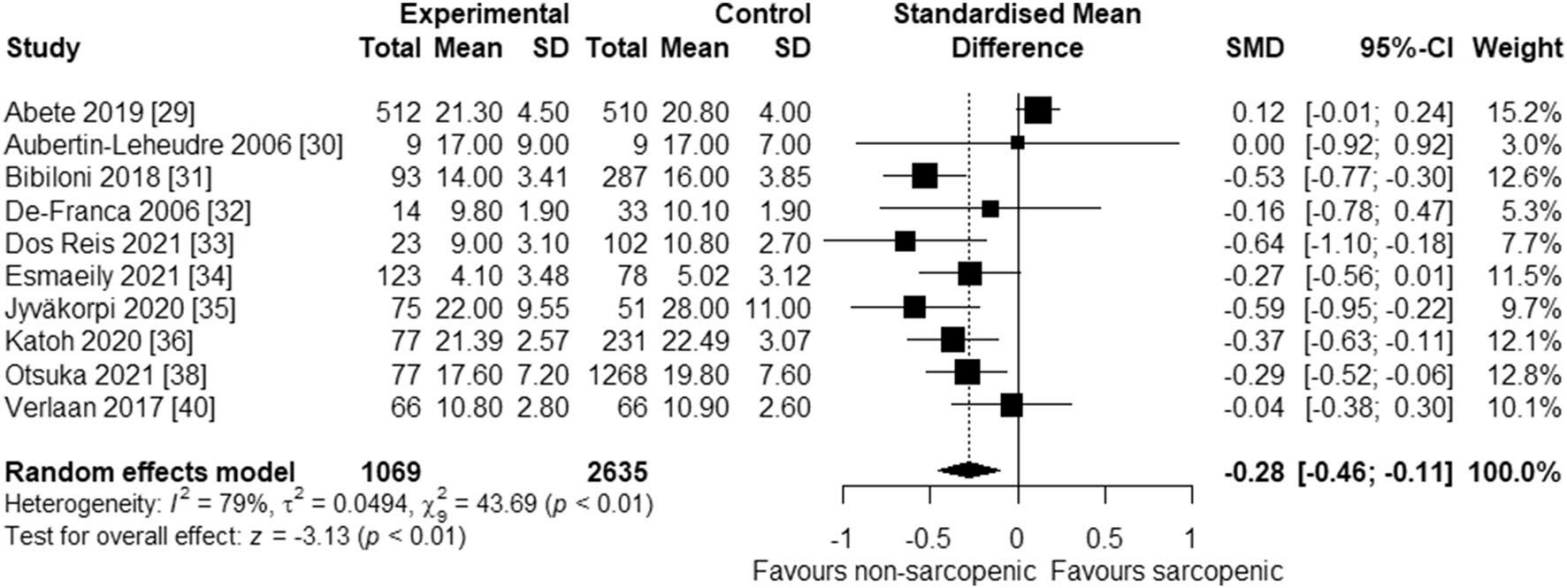
Forest plot showing the effect of dietary or circulatory levels of MUFA on sarcopenia in 10 observational studies. SD, standard deviation; SMD, standardized mean difference; CI, confidence interval

**Fig. 3:**
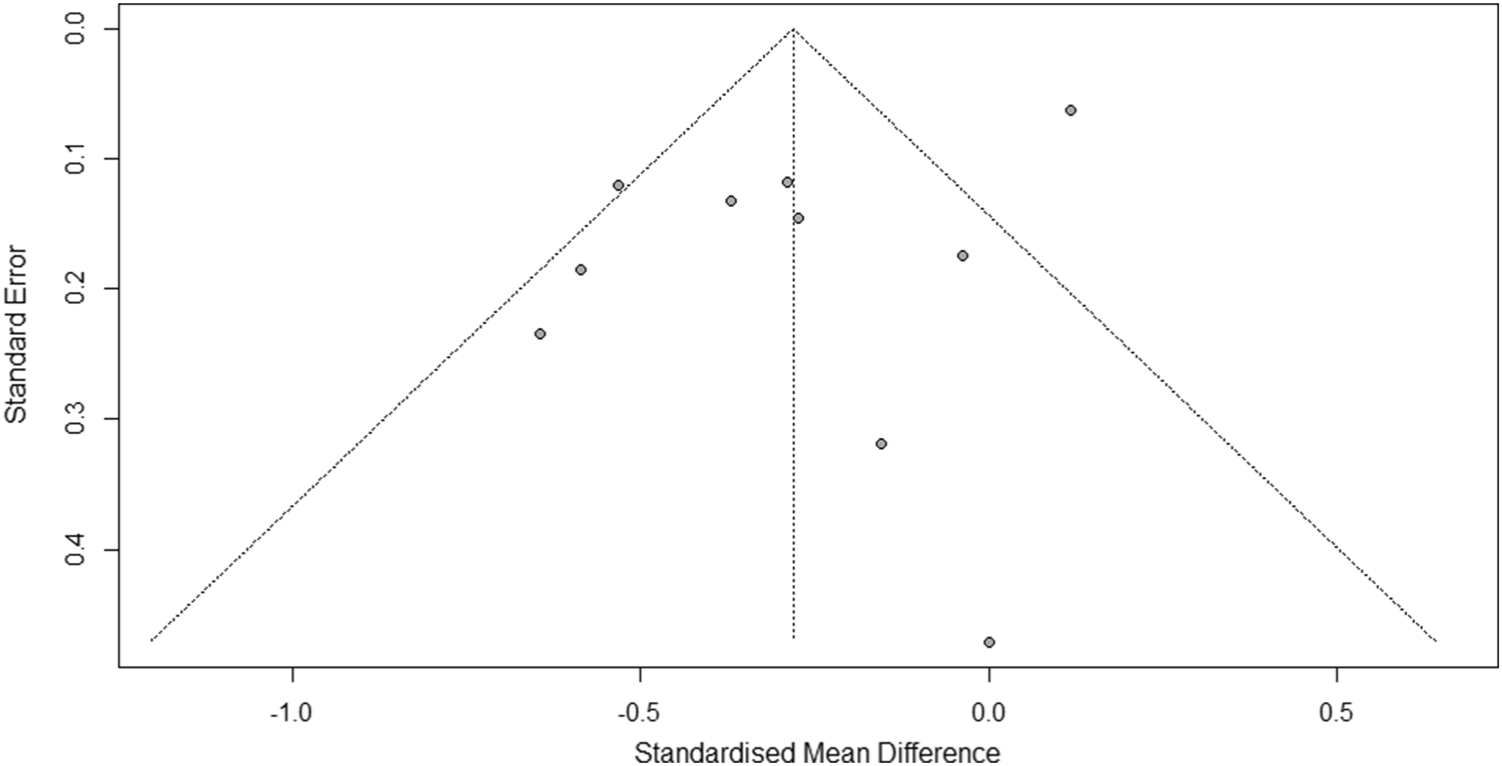
Funnel plot for publication bias

Ten of the 12 studies were included in the meta-analysis as the required pooling data were available [29–36, 38, 40]. These studies involved a total of 3704 participants. The overall combined SMD showed that the dietary or circulatory MUFA level is inversely associated with the risk of sarcopenia (SMD = − 0.28, 95% CI − 0.46 to − 0.11; *p* < 0.01) (Fig. 2). A high level of heterogeneity was found among the studies (*I*^2^ = 79%, *p* < 0.01). The funnel plots showed no evidence of publication bias in the included studies (Fig. 3). Likewise, Egger’s and Begg’s rank tests detected no evidence of publication bias (*p* = 0.09 and *p* = 0.7, respectively).

The results of the subgroup analyses are shown in Table 3. The pooled analysis of three studies that involved 1087 subjects with sarcopenic obesity did not show a significant association between MUFA intake and sarcopenic obesity (SMD = 0.11, 95% CI − 0.01 to 0.22, *p* = 0.08). Eight studies on 3532 people over 65 years of age confirmed a significant effect of MUFA on sarcopenia (SMD = − 0.26, 95% CI − 0.45 to − 0.07, *p* < 0.01). The remaining two studies involved 172 subjects with an overall mean age less than 65 years, and the pooled analysis showed a negative, although not significant, association between MUFA levels and sarcopenia (SMD = – 0.45, 95% CI − 0.92 to 0.02, *p* = 0.06). Likewise, eight studies included both men and women and showed a negative association (SMD = – 0.26, 95% CI − 0.45 to − 0.07, *p* < 0.01). Regarding the diagnostic criteria of sarcopenia, the significant result existed in EWGSOP2 (SMD = – 0.46, 95% CI − 0.71 to − 0.21; *p* < 0.01) and AWGS (SMD = – 0.33, 95% CI − 0.50 to − 0.15; *p* < 0.01), but lost in others (SMD = – 0.09, 95% CI − 0.02 to 0.20; *p* = 0.12). The above findings were also confirmed in dietary MUFA levels (SMD = – 0.27, 95% CI − 0.47 to − 0.07, *p* < 0.01), considering dietary recall tools (SMD = – 0.37, 95% CI − 0.55 to − 0.19, *p* < 0.01). Additionally, MUFA levels expressed as a percent of total energy (SMD = – 0.29, 95% CI − 0.57 to − 0.01, *p* = 0.04), grams/day (SMD = – 0.28, 95% CI − 0.48 to − 0.08, *p* < 0.01), and MUFA/SFA ratio (SMD = – 0.34, 95% CI − 0.57 to − 0.12, *p* < 0.01) showed significant negative associations between MUFA and sarcopenia.

**Table 3:** Subgroup analyses on the effects of MUFA on sarcopenia

	No. of studies	No. of participants	*I*^2^ (%)	SMD (95% CI)	*p*-value
Overall	10	3704	79.0	– 0.28 (– 0.46, – 0.11)	< 0.01
Sarcopenic obesity	3	1087	0.0	0.11 (– 0.01, 0.22)	0.08
Age					
≥ 65 years	8	3532	82.0	– 0.26 (– 0.45, – 0.07)	< 0.01
< 65 years	2	172	34.0	– 0.45 (– 0.92, 0.02)	0.06
Gender					
Women	1	18	–	0.00 (– 0.92, 0.92)	–
Men	1	126	–	– 0.59 (– 0.95, – 0.22)	–
Women and Men	8	3560	82.0	– 0.26 (– 0.45, – 0.07)	< 0.01
Sarcopenia classification					
Confirmed/severe sarcopenia	7	3028	42.0	– 0.26 (– 0.41, – 0.11)	< 0.01
Probable sarcopenia	2	300	46.0	– 0.41 (– 0.74, – 0.08)	0.01
Sarcopenia diagnostic tool					
EWGSOP	1	380	–	– 0.53 (– 0.77, – 0.30)	–
EWGSOP2	3	452	26.0	– 0.46 (– 0.71, – 0.21)	< 0.01
AWGS	2	1653	0.0	– 0.33 (– 0.50, – 0.15)	< 0.01
Others	4	1219	0.0	– 0.09 (– 0.02, 0.20)	0.12
Exposure assessment					
Dietary	9	3396	80.0	– 0.27 (– 0.47, – 0.07)	< 0.01
FFQ	2	1223	84.0	– 0.06 (– 0.44, 0.32)	0.6
Dietary recall	7	2173	34.0	– 0.37 (– 0.55, – 0.19)	< 0.01
Circulatory	1	308	–	– 0.37 (– 0.63, – 0.11)	–
MUFA intake expressed as					
Percent of total energy	6	1832	87.0	– 0.29 (– 0.57, – 0.01)	0.04
Percent of total FA	1	308	–	– 0.37 (– 0.63, – 0.11)	–
Grams/day	3	1488	0.0	– 0.28 (– 0.48, – 0.08)	< 0.01
MUFA/SFA ratio	2	327	0.0	– 0.34 (– 0.57, – 0.12)	< 0.01

*MUFA* monounsaturated fatty acids, *SMD* standardized mean difference, *EWGSOP* the European Working Group on Sarcopenia in Older People, *EWGSOP2* revised EWGSOP, *AWGS* the Asian Working Group for Sarcopenia, *FFQ* food frequency questionnaires, *FA* fatty acids, *SFA* saturated fatty acids

## Discussion

Despite the limited number of observational studies included in our systematic review and meta-analysis, the results suggest that MUFA intake is inversely associated with the risk of sarcopenia. In addition, the corresponding result subsisted in several subgroup analyses that were performed based on population characteristics, MUFA assessment tools, and sarcopenia diagnosis. Among the 10 studies that were meta-analysed, six studies reported a significantly lower MUFA intake in sarcopenic subjects compared to non-sarcopenic ones [31, 33–36, 38]. On the other hand, the remaining four studies did not report any significant difference in MUFA intake between sarcopenic and control participants [29, 30, 32, 40]. Furthermore, two more studies that were included in the review but not in the meta-analysis did not find a significant association between MUFA intake and the risk of sarcopenia [37, 39]. Montiel-rojas 2020 reported that substituting SFAs by MUFAs does not affect sex-specific sarcopenia risk score in 986 older European adults [37]. Similarly, ter Borg 2018 found no significant associations between blood MUFA status and sarcopenia in 227 community-dwelling older adults [39]. Therefore, although MUFA intake showed a negative association with sarcopenia in our meta-analysis, the available data are still insufficient to confirm this association, and further investigations are required.

Our finding that a higher intake of MUFA may have positive effects on sarcopenia is consistent with previous reports indicating that MUFA may inhibit muscle atrophy by enhancing mitochondrial oxidative ability, protein synthesis, insulin sensitivity, and reducing inflammation [41]. For example, MUFA have been reported to prevent SFA-induced insulin resistance and exert anti-inflammatory effects in skeletal muscle cells [42–44]. MUFA protects against SFA-induced inflammation and insulin resistance by promoting triglyceride accumulation and mitochondrial beta-oxidation through peroxisome proliferator-activated receptor-alpha (PPAR-α) and protein kinase A-dependent mechanisms [43]. Likewise, OL inhibits SFA-mediated activation of the mammalian target of rapamycin complex 1 (mTORC1)/p70S6K, leading to a reduction in intracellular inflammatory signalling and cytotoxicity [45]. Additionally, in aging individuals, there is an elevation of pro-inflammatory cytokines, such as tumor necrosis factor-alpha (TNF-α), interleukin 6 (IL-6) and C-reactive protein (CRP), that contribute to low muscle mass and strength [46]. MUFA, such as OL is shown to reduce serum CRP and increase anti-inflammatory gene expression [47, 48]. These anti-inflammatory actions of MUFA in skeletal muscle cells may contribute to their ability to preserve muscle mass and function [41]. MUFA also prevents muscle atrophy and increase muscle differentiation by enhancing mitochondrial function and reducing mitochondrial ROS [49, 50]. It is demonstrated that an OL-enriched diet may increase muscle protein synthesis, associated with increased expression of genes implicated in stimulating mitochondrial β-oxidation including PPAR-α and PPAR-beta (PPAR-β), as well as carnitine palmitoyl transferase 1 beta (CPT-1β) [51, 52]. Therefore, MUFA intake, particularly OL, may have the potential as an effective nutrition-based preventive or therapeutic strategy against sarcopenia.

Of the 10 studies included in our meta-analysis, three studies involved subjects with sarcopenic obesity [29, 30, 32]. Sarcopenic obesity is defined as the presence of both obesity (high body fat percentage or fat mass index) and sarcopenia [53]. It is associated with an increased risk of cardiovascular disease and mortality in the aging population [54]. Sarcopenic obesity is proposed to be considered a distinctive clinical condition because its effect on health and clinical outcomes is different from those related to obesity or sarcopenia. Due to the negative interaction between fat mass accumulation and low muscle mass/function, sarcopenic obese subjects have a higher risk for metabolic disorders and functional declines compared to sarcopenic or obese individuals. The management of sarcopenic obesity is challenging and preventive/treatment strategies may not only be a combination of the methods used to manage obesity and sarcopenia. It is suggested that personalized multidisciplinary approaches with dietary, physical, psychological, and pharmacological mechanisms may be ideal to manage this condition [53]. Beside the effects of MUFAs on muscle health, previous studies indicate that MUFAs may accelerate overall lipid oxidation in peroxisome and mitochondria by stimulating peroxisomal and mitochondrial beta-oxidation [43, 55]. Long-chain MUFAs can also improve obesity-related metabolic dysfunction by upregulation of PPAR-gamma (PPAR-γ) and downregulation of inflammatory markers in the adipose tissue [56]. Indeed, the anti-inflammatory effect of MUFA-rich Mediterranean diet is described to decrease the risk of obesity-related metabolic syndrome [57]. Nevertheless, our analysis of three studies involving 1087 participants did not show a significant association between MUFA intake and sarcopenic obesity (Table 3).

Although the number of studies with sex descriptions is limited, some sex-related differences in experimental models are described to be associated with the effect of diet on sarcopenia [58]. The majority of the studies included in our review were designed for both men and women and sex-specific analyses were not performed. The pooled analysis of these studies showed that MUFA levels are significantly lower in sarcopenic subjects compared to the non-sarcopenic ones (Table 3). On the other hand, the study by Jyväkorpi et al. examined community-living oldest old men, and Aubertin-Leheudre et al. studied a group of sarcopenic and non-sarcopenic obese post-menopausal women [30, 35]. Jyväkorpi et al. reported that higher MUFA intake and MUFA/SFA ratio are both associated with lower sarcopenia risk [35]. In contrast, the results of the Aubertin-Leheudre et al. study did not show any significant difference in MUFA intake among the two groups [30]. In this study, however, the non-sarcopenic women had a greater body mass index (BMI), abdominal fat mass, and visceral fat mass than the sarcopenic women [30]. The BMI and body fat are reported to be associated with sarcopenia [59, 60]. In addition, Aubertin-Leheudre et al. included 22 women, suggesting that a large sample-sized study may be more reliable to address the correlation [30].

The articles included in our study were published between 2006 and 2021, involving a period with considerable changes in the diagnostic criteria of sarcopenia. Sarcopenia was first described in 1989 as an age-related reduction in muscle mass [1, 2]. In 2000, sarcopenia was defined as two SD below the mean value of appendicular skeletal muscle mass divided by height squared of sex-specific young adult reference value [61]. Other definitions of sarcopenia later emerged that required measurement of a combination of three parameters, which are muscle mass, muscle strength, and physical performance. In 2010, the EWGSOP proposed an operational definition of sarcopenia that added muscle function to previous definitions based on low muscle mass [6]. According to the EWGSOP, the diagnosis of sarcopenia requires the detection of low muscle mass with low muscle function (muscle strength or physical performance). The EWGSOP was then updated in 2019 as EWGSOP2 [7]. The EWGSOP2 used poor muscle strength as the primary parameter of sarcopenia instead of low muscle mass. According to the EWGSOP2, sarcopenia is probable when low muscle strength is detected, sarcopenia diagnosis is confirmed when low muscle quantity and quality are identified, and sarcopenia is severe when low muscle strength, low muscle quantity/quality and poor physical performance are detected altogether. Although the EWGSOP did not advise specific cut-off points, the EWGSOP2 provided clear cut-off points of the parameters used for sarcopenia diagnosis [6, 7]. Furthermore, in 2014, the AWGS was proposed, which has a similar definition as the EWGSOP and was later revised in 2019 [28]. The AWGS established an EWGSOP-based consensus that denoted cut-off points for diagnostic variables of sarcopenia in Asian populations. When we performed a subgroup analysis by the diagnostic criteria of sarcopenia, the corresponding result existed in EWGSOP2 and AWGS but was lost in the other diagnostic criteria. In addition, we observed a significant decrease in heterogeneity in this subgroup analysis (Table 3).

This systematic review and meta-analysis have some limitations. First, there was a substantial level of heterogeneity in the meta-analysis, which could be due to the heterogeneity in the methods and diagnostic criteria used to define sarcopenia among the included articles. Second, previous evidence suggests that there is sex-specific differences in pathophysiological mechanisms and risk factors for sarcopenia [62, 63]. However, many of the included studies did not consider sex differences, and therefore, a subgroup analysis by sex could not be performed in these studies. Another limitation of our study is that due to scarcity of data, we were unable to perform a subgroup analysis based on co-diseases present in the population, which could certainly affect sarcopenia. Finally, there is a limitation of relevant literature, in which 12 studies were identified and only 10 of them could be meta-analysed. Our study also has several strengths. First, to our knowledge, this is the first meta-analysis of observational studies on the relationship between dietary or circulatory MUFA levels and sarcopenia based on a comprehensive literature search. In addition, almost all of the included studies were published in recent years, suggesting a potential novel topic in our systematic review and meta-analysis.

Overall, this systematic review and meta-analysis provide preliminary evidence that MUFA intake is different among people with sarcopenia compared to people without sarcopenia. However, current evidence is still insufficient to demonstrate the definite relationship between MUFA intake and sarcopenia. More well-designed prospective cohort studies along with clinical trials are still needed.

## Supplementary Information

Below is the link to the electronic supplementary material.

Supplementary file1 (DOCX 18 KB)

## Data Availability

From authors upon request.
