# Supplementary material for: Relationship between monounsaturated fatty acids and sarcopenia: a systematic review and meta-analysis of observational studies
Source: Aging Clin Exp Res. 2023 Jun 21;35(9):1823–34. doi: 10.1007/s40520-023-02465-0 (PMC10460305; doi:10.1007/s40520-023-02465-0)
Supplement: Supplementary file 1 — Supplementary file1 (DOCX 18 KB) [file 40520_2023_2465_MOESM1_ESM.docx]

| **Table S1.** Search queries used to search in PubMed, Scopus, and Web of Science databases | | | |  |
| --- | --- | --- | --- | --- |
|  | **PubMed** | **Scopus** | **Web of Science** | |
| **Free text terms / natural language terms** | ("monounsaturated fatty acid*"[All Fields] OR "monounsaturated fat*"[All Fields] OR "mufa*"[All Fields] OR "palmitoleic acid"[All Fields] OR "palmitoleic"[All Fields]) OR "oleic"[All Fields]) AND ("sarcopenia"[MeSH Terms] OR "sarcopenia"[All Fields] OR "sarcopenic"[All Fields] OR "muscular atrophy"[All Fields] OR "muscle strength"[All Fields] OR "physical performance"[All Fields] OR "muscle mass"[All Fields]) AND (english[Filter])  **Search result = 60** | ( ALL ( "Monounsaturated fatty acid*" ) OR ALL ( "Monounsaturated fat*" ) OR ALL ( mufa* ) OR ALL ( palmitoleic ) OR ALL ( oleic ) ) AND ( TITLE-ABS-KEY ( sarcopenia ) OR TITLE-ABS-KEY ( sarcopenic ) OR TITLE-ABS-KEY ( "muscular atrophy" ) OR TITLE-ABS-KEY ( "muscle strength" ) OR TITLE-ABS-KEY ( "physical performance" ) OR TITLE-ABS-KEY ( "muscle mass" ) ) AND ( LIMIT-TO ( DOCTYPE , "ar" ) OR LIMIT-TO ( DOCTYPE , "ed" ) OR LIMIT-TO ( DOCTYPE , "le" ) ) AND ( LIMIT-TO ( LANGUAGE , "English" ) )  **Search result = 284** | (((((ALL=("monounsaturated fatty acid*")) OR ALL=("monounsaturated fat*")) OR ALL=(MUFA*)) OR ALL=(palmitoleic)) OR ALL=(oleic)) AND (((((((((ALL=(sarcopenia)) OR ALL=( sarcopenic )) OR ALL=("muscular atrophy")) OR ALL=("muscle strength")) OR ALL=("hand strength")) OR ALL=("pinch strength")) OR ALL=("handgrip strength")) OR ALL=("gait speed")) OR ALL=("muscle mass"))  **Search result = 54** | |
| **Controlled vocabulary** **terms / Subject terms**  (MeSH terms, INDEXTERMS) |  | ( INDEXTERMS ( "monounsaturated fatty acids" ) ) AND ( INDEXTERMS ( sarcopenia ) )  **Search result = 16** | Web of Science does not have controlled vocabulary search. | |

| Table S2. Risk of bias assessment | | | | | |
| --- | --- | --- | --- | --- | --- |
| Study | Selection | Comparability | Outcome | Total No. stars | Quality of study |
| Abete 2019 [29] | ******* | ****** | ****** | 7 | High |
| Aubertin-Leheudre 2006 [30] | ******* | ***** | ****** | 6 | Moderate |
| Bibiloni 2018 [31] | ******* | ****** | ****** | 7 | High |
| De-franca 2020 [32] | ******* | ****** | ****** | 7 | High |
| Dos Reis 2021 [33] | ******* | ****** | ****** | 7 | High |
| Esmaeily 2021 [34] | ******* | ****** | ****** | 7 | High |
| Jyvakorpi 2020 [35] | ******* | ****** | ****** | 7 | High |
| Katoh 2020 [36] | ******* | ****** | ****** | 7 | High |
| Montiel-rojas 2020 [37] | ******* | ****** | ****** | 7 | High |
| Otsuka 2021 [38] | ******* | ****** | ****** | 7 | High |
| ter Borg 2018 [39] | ******* | ****** | ****** | 7 | High |
| Verlaan 2017 [40] | ******* | ****** | ******* | 8 | High |
